# Bandwidth Improvement in Ultrasound Image Reconstruction Using Deep Learning Techniques

**DOI:** 10.3390/healthcare11010123

**Published:** 2022-12-30

**Authors:** Navchetan Awasthi, Laslo van Anrooij, Gino Jansen, Hans-Martin Schwab, Josien P. W. Pluim, Richard G. P. Lopata

**Affiliations:** 1 Photoacoustics and Ultrasound Laboratory Eindhoven (PULS/e), Department of Biomedical Engineering, Eindhoven University of Technology, 5612 AZ Eindhoven, The Netherlands; 2 Medical Image Analysis Group (IMAG/e), Department of Biomedical Engineering, Eindhoven University of Technology, 5612 AZ Eindhoven, The Netherlands; 3 Department of Biomedical Engineering and Physics, Amsterdam University Medical Center, 1105 AZ Amsterdam, The Netherlands; 4 Image Sciences Institute, University Medical Center, 3584 CX Utrecht, The Netherlands

**Keywords:** ultrasound imaging, bandwidth (BW) improvement, convolutional neural networks, image reconstruction, deep learning

## Abstract

Ultrasound (US) imaging is a medical imaging modality that uses the reflection of sound in the range of 2–18 MHz to image internal body structures. In US, the frequency bandwidth (BW) is directly associated with image resolution. BW is a property of the transducer and more bandwidth comes at a higher cost. Thus, methods that can transform strongly bandlimited ultrasound data into broadband data are essential. In this work, we propose a deep learning (DL) technique to improve the image quality for a given bandwidth by learning features provided by broadband data of the same field of view. Therefore, the performance of several DL architectures and conventional state-of-the-art techniques for image quality improvement and artifact removal have been compared on in vitro US datasets. Two training losses have been utilized on three different architectures: a super resolution convolutional neural network (SRCNN), U-Net, and a residual encoder decoder network (REDNet) architecture. The models have been trained to transform low-bandwidth image reconstructions to high-bandwidth image reconstructions, to reduce the artifacts, and make the reconstructions visually more attractive. Experiments were performed for 20%, 40%, and 60% fractional bandwidth on the original images and showed that the improvements obtained are as high as 45.5% in RMSE, and 3.85 dB in PSNR, in datasets with a 20% bandwidth limitation.

## 1. Introduction

Ultrasound (US) imaging is the modality of choice for many diagnostic purposes [1,2]. US is a cost-effective, versatile, and non-invasive imaging modality and is therefore often applied for various imaging tasks, such as cardiac imaging [3], abdominal imaging [4] or obstetrics [5] in addition to many other applications. The amount of bandwidth (BW) necessary for imaging varies from task to task [6]. The frequency range of the transducer is a key factor in determining the resolution for B-mode imaging [7,8,9]. The temporal bandwidth of the ultrasound transducer is directly associated with the image resolution and image quality. A large bandwidth therefore ensures that the smallest possible detail is visible in both axial and lateral direction. Certain tasks require a higher bandwidth transducer to image structures that are normally not clearly distinguishable with lower bandwidth transducers, as in the case of intra-vascular microvessel imaging [10]. Intravascular ultrasound (IVUS) based methods require high-frequency transducers which are not available commercially for imaging the microvessels [10]. High-frequency transducers are also required for imaging in various clinical applications and in small animal studies for scanning rats, mice and zebrafish [11,12]. Furthermore, for intra-vascular or intra-cardiac US techniques, the responsiveness of the PZT crystals (with certain thickness) with respect to frequency also limits the bandwidth [13]. The improvement in reconstruction that transforms low bandwidth image reconstruction to high bandwidth image reconstruction can potentially help in making these tasks possible with lower bandwidth transducers. Traditional methods for bandwidth improvement in US already exist; for example, in ultrafast imaging, the bandwidth of the transducer limits the image quality of the reconstruction. Resolution enhancement compression (REC) is then utilized for enhancing the bandwidth [14]. In Ref. [15], a pre-filtering technique was used to increase the output signal bandwidth for the US transmit-receive systems. These methods are based on traditional approaches but are not data driven and hence do not consider the probability distributions of actual US.

In this work, we proposed a new technique for bandwidth improvement based on deep learning (DL). DL has already been successful in addressing a wide range of problems, such as speech recognition [16], language processing [17], image classification [18] and image segmentation [19]. Furthermore, DL has been used in various medical applications [20,21,22] like Computed Tomography (CT) imaging [23,24], US imaging [25,26,27,28], and MRI imaging [29,30] to improve image quality. DL has applications in denoising the image, image analysis and super-resolution. In super-resolution, a low-resolution image is transformed into a higher-resolution image. This task of bandwidth enhancement in ultrasound is similar to super resolution problems in computer vision. In ultrasound imaging, DL methods have been applied for various kinds of image enhancement tasks. Perdios et al. [31] proposed a residual CNN based method, using multiscale and multichannel filtering properties, for transforming a low-quality estimate to a high-quality image for ultrafast US imaging. In the work by Yoon et al. [32], a DL-based model is created that can transform a sub-sampled radiofrequency (RF) signal directly to a normal-quality B-mode image. However, none of these tasks have actively targeted bandwidth improvement.

In this work, we assess the performance of several popular DL architectures for the first time on five different US datasets consisting of B-mode images. The three model architectures compared are: super resolution convolutional neural network (SRCNN) [33], U-Net [34], and a Residual Encoder Decoder network (REDNet) architecture based on the work by Gholizadeh-Ansari et al. [35]. We also introduce two loss functions namely, PL1 (scaled mean square error) and PL2 (a combination of mean square error (RMSE) and perceptual loss), to improve the results for these different architectures. To show that these models really improve the reconstruction and bandwidth, we also compare these methods with conventional histogram-based methods for contrast enhancement. For this, we use histogram equalization (HE) and contrast limited adaptive histogram equalization (CLAHE). The main contributions of the work can be summarized as follows:

- A DL-based method is proposed for directly transforming a low-bandwidth image reconstruction to a broadband image reconstruction. The transducers are band-limited in nature. The band-limitation of the transducer directly affects the resolution of the US image. This DL based technique can help in transforming the low-bandwidth image reconstruction obtained into a high-bandwidth image reconstruction, which can help in removing artifacts, improving resolution and image quality in general.
- A data driven method was proposed for bandwidth improvement in US imaging for the first-time using DL. The DL model proposed is light weight, has low computational complexity, and can be utilized in real settings. Error analysis, frequency domain comparisons and speckle characteristic comparisons were performed to show the benefits of the proposed method as compared with other state-of-the-art networks.
- A scaled mean square error loss for the training of the DL network is introduced to solve the vanishing gradient problem. The models were trained on only one set of training data and tested on five different datasets with different properties to show the generalizable and scalable property of the proposed model in real settings.

The rest of this article is organized as follows. In Section 2, we briefly introduce the deep learning methods compared in this work, training strategy, and loss functions. In Section 3, datasets and experiments have been discussed, whereas in Section 4, details about the figures of metric have been presented. In Section 5, results are discussed, while Section 6 details about the discussion, analysis of all the models and Section 7 concludes the work.

## 2. Material and Methods

We evaluate the proposed DL-based methods along with the two different loss functions, for enhancing the bandwidth of the band-limited US images.

### 2.1. Deep Learning Techniques

#### 2.1.1. SRCNN

**Figure 1:**
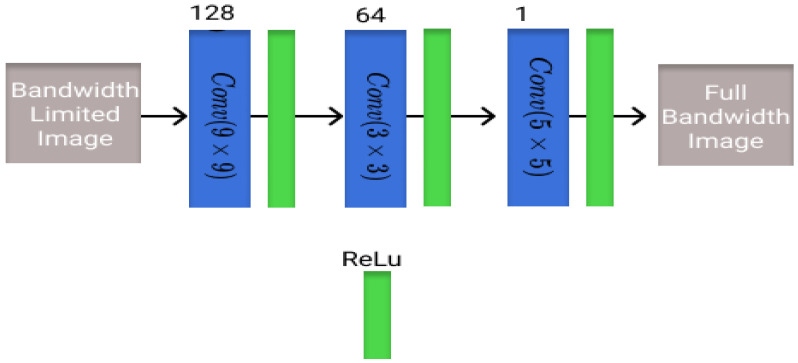
Visualization of the SRCNN architecture utilized in this study with different blocks and the number of filters present in each layer.

The first architecture that is evaluated is known as the super-resolution convolutional neural network (SRCNN) and is one of the first models to learn end-to-end mapping of low-resolution to high-resolution images. It was one of the first super-resolution DL architectures for computer vision images [33]. The model architecture is shown in Figure 1 with corresponding blocks and the number of filters in each layer.

#### 2.1.2. UNet

**Figure 2:**
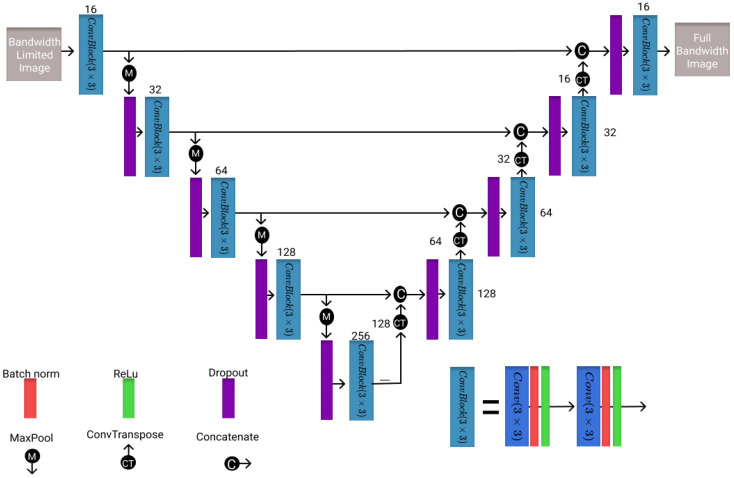
Visualization of the U-Net architecture utilized in this study with different blocks and the number of filters present in each layer.

The second architecture evaluated is the U-Net, which is often used for medical imaging segmentation problems [34]. However, it has also been applied in the field of super resolution and denoising to improve image quality or resolution [36,37]. Therefore, the U-Net model is chosen as the benchmark model. However, a ReLu activation instead of the sigmoid activation is applied here for bandwidth improvement. Thus, the model is slightly adjusted by removing the sigmoid activation in the last layer. The model architecture is shown in Figure 2 with the number of filters in each layer and the corresponding block representations.

#### 2.1.3. REDNet

**Figure 3:**
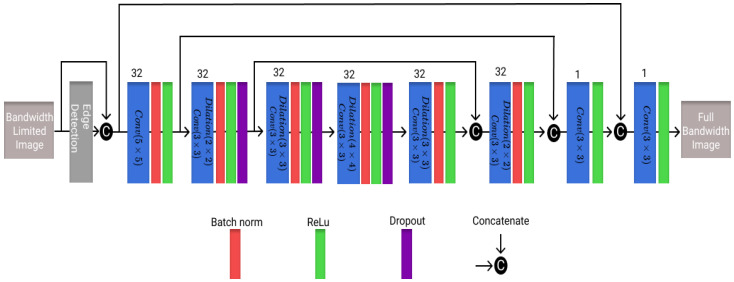
Visualization of the REDNet architecture utilized in this work with different blocks and the number of filters present in each layer.

The third architecture utilized here is a residual encoder decoder network (REDNet), which was originally developed to transform low-dose CT images to high-dose CT images [35]. As can be seen in Figure 3, the architecture uses an edge detection layer that uses a Sobel filter for feature extraction. The model uses dilated convolutional layers to increase the receptive field of the model as this is more computationally efficient than increasing either the filters or the kernel size [38]. As a small adjustment to the model, dropout layers with a value of 0.05 were added to reduce over-fitting. The model architecture is shown in Figure 3, with the number of filters in each layer and corresponding block representations.

### 2.2. Loss Functions

We evaluated the models for two different loss functions:

- PL1 loss: Scaled mean square error loss. PL1 loss is used as it is more sensitive towards outliers and is the most used loss function [39]. The network was trained using the proposed PL1 (scaled-MSE) loss function, defined as:
  (1) $$L=\frac{1}{N}\sum_{i=1}^{N}{∥y_{model}-y_{FB}\left(i\right)∥}^{2}\times \tau$$
  where, $y_{model}$ are the predictions of the model, $y_{FB}\left(i\right)$ are the true signals and τ is the scaling factor [40].
- PL2 loss: Recently perceptual loss has gained wide popularity as it can extract features, and can compare the content, style, and high-level differences between images [35]. Hence, we used PL2 loss as the combination of RMSE and perceptual loss. The losses are combined in a ratio of 30:70 [35].

### 2.3. Model Specifications

The models are implemented in TensorFlow [41] using Keras [42] as back-end. All models are trained using a batch size of 16 and run for either 300 epochs or until the validation loss stops improving. For each image, 64 image patches are made with each at a size of 128 × 128 by randomly selecting overlapping patches of 128 × 128 from the image. This means that 25,600 image patches are used to train the models and 8640 image patches are used to validate the models. The Adam optimizer [43] is used with the following parameters: Learning rate (lr) = $1\;\cdot \;10^{-4}$, $\beta_{1}$ = 0.9, $\beta_{2}$ = 0.999, ϵ = None and decay = 0. The models are trained only for 20% bandwidth limitation using the Phantom I dataset and tested on other datasets (Table 1) and bandwidth scenarios. This is to show the generalization of the model on different datasets and bandwidth scenarios.

## 3. Datasets and Experiments

### 3.1. Datasets

We have acquired five different phantom or in vitro datasets for our experiments, the details of which are explained below. Table 1 gives the distribution of the images in the dataset for training, validation, and testing purposes. A Verasonics Vantage research system (Verasonics, Kirkland, WA, USA) was employed for acquisition of the data. With a Verasonics L11-5v linear array transducer, 101 Plane Waves were transmitted at equi-spaced angles, ranging from $-25^{\circ }$ to $25^{\circ }$. After careful consideration, the range was reduced to $-15^{\circ }$ to $15^{\circ }$ (61 PWs, step of $0.5^{\circ }$) in post-processing to reduce grating lobe [44]. The data that support the findings of this study are available on request from the corresponding author.

#### 3.1.1. Phantom I

A Bloom 300 (8%) gelatin solution was infused with high concentrations of silicon carbide (SiC) scatterers [45]. Three different batches with different concentrations of SiC: 1 g/dL, 8 g/dL, and 16 g/dL were produced for this dataset. After solidification, the latter two batches were diced into small cubes of roughly 1 cm^3^ and embedded in the first batch, which was still fluid. In addition, fresh mandarin segments and raspberries were added to provide some complex structures in the image data. A total of 669 US images using the full bandwidth were acquired and were limited to different fractional bandwidths. The images were split into a training, validation, and test set of 400, 135, and 134, respectively, dividing the data into a 60, 20, and 20 split (Table 1).

#### 3.1.2. Phantom II

Based on previous experience, a second multi-purpose phantom was created that consists of only canned mandarins embedded in gelatin with a lower concentration of SiC scatterers (0.5 g/dL). Mandarins were processed to remove the connective layers and membranes, that would otherwise absorb or reflect a lot of the acoustic energy. A total of 90 images were used for testing the model developed for bandwidth improvement (Table 1).

#### 3.1.3. Commercial Phantom

This phantom is a commercially available phantom (Model 539, Multipurpose US Phantom, CIRS, Inc., Norfolk, VA, USA) for quantitative US quality measurements. The phantom provides a combination of tissue mimicking target structures of varying sizes and contrasts and monofilament line targets for distance measurements. This dataset consists of 31 US images acquired at full available bandwidth (3.04 MHz passband) and derived datasets that were band-limited to the following fractional bandwidths, i.e., 20%, 40%, and 60% (Table 1).

#### 3.1.4. Carotid Artery

Porcine carotid arteries (CAs), after removing most of the surrounding tissue, were embedded in the low concentration (0.5 g/dL) scattering emulsion. The arteries were tied up on both ends and pressurized with water. This dataset consists of 70 full bandwidth US images of two porcine carotid arteries, which were limited to different fractional bandwidth ranges, i.e., 20%, 40%, and 60% (Table 1).

#### 3.1.5. In Vivo Carotid Mimicking Setup

For this phantom, another two carotid arteries were prepared identically. This time however, they were embedded in skin-on muscle and adipose tissue of the porcine abdomen and neck. Cuts were made skin side down. These cuts were approximately half the thickness of the tissue in depth. The arteries were put inside of those cuts. This dataset consists of 239 images and was only used for testing the model (Table 1).

Various experiments were performed for three different band-limited scenarios (20%, 40%, and 60% bandwidth) and for the five different test datasets to show the effectiveness of the proposed techniques for improving the frequency content of the band-limited images. A bandwidth limitation of 20% means that the frequencies of the target band-limited image are further reduced to only 20% and used as the input for the DL-based model. The reason of choosing these specific bandwidths is as follows: 60% bandwidth limitation is quite realistic while 40% and 20% limitation is to check the limit of bandwidth limitation which can be used for US imaging. All images in the datasets were processed in MATLAB (Release 2019a, The MathWorks, Inc., Natick, MA, USA) to attain image pairs of limited bandwidth and full bandwidth reconstructions. The RF data are band-filtered. From these processed RF data, the images are reconstructed for band-limited as well as full bandwidth images. Envelope detection and log compression is applied to the resulting RF images to retrieve B-mode data. The images obtained have a size of 801 × 401 pixels.

### 3.2. Experiments

We compared the DL-based methods proposed with conventional HE-based methods.

#### 3.2.1. Histogram Equalization (HE)

HE has been widely used as a method for enhancing the contrast of images in various applications, such as radar image processing as well as medical image processing [46,47]. HE flattens the density distribution of the image and enhances its contrast [48,49]. There are some disadvantages to this technique such as artifacts, and deterioration of the visual quality; thus, HE does not always give improved results and can lead to an enhanced noise level [50].

#### 3.2.2. Contrast Limited Adaptive Histogram Equalization (CLAHE)

CLAHE is another histogram equalization technique and consists of the following four steps [51]:

- Partitioning of the image into non-overlapping and continuous patches.
- Clipping the histogram of each patch above a threshold and distributing the pixels to all the gray values.
- Applying HE on each patch.
- Interpolating the mapping between separate patches.

Although CLAHE usually performs better than HE, it also suffers from noise enhancement and providing no one-to-one correspondence between the input and the enhanced image [51]. Please refer to Ref. [51] for more details about the method.

## 4. Figures of Metric

The reconstructions obtained using the different techniques were evaluated using the Root Mean Square Error (RMSE), Peak Signal to Noise Ratio (PSNR), and Pearson Correlation (PC) for all the methods.

### 4.1. RMSE

RMSE is the root of the mean squared difference between the pixel intensities of two images [52]:

(2) $$\mathrm{RMSE}=\sqrt{\frac{\sum_{i=1}^{n}{\left(y_{model}\left(i\right)-y_{FB}\left(i\right)\right)}^{2}}{n}}$$

Equation (2) gives the RMSE metric with $y_{model}$ being the image generated by the technique, $y_{FB}$ being the full bandwidth image and *n* being the number of pixels of both images. RMSE is sensitive towards the outliers and is a measure of accuracy of the model. This metric helps in quantifying the error between the reconstructions obtained as compared to the full-bandwidth reconstructions.

### 4.2. PSNR

PSNR is the ratio between the maximum value in the full bandwidth image and the mean square error between the model predicted image and the full bandwidth image [53].

(3) $$\mathrm{PSNR}=10\log_{10}\left(\frac{I_{max}^{2}\left(y_{FB}\right)}{MSE(y_{model},y_{FB})}\right)$$

Equation (3) gives the PSNR metric with $I_{max}$ the maximum pixel intensity, $y_{FB}$ the full bandwidth image, and $y_{model}$ the image generated by the technique, where the mean square error (MSE) is the square of the RMSE. PSNR is often used for quantifying the reconstruction quality [37].

### 4.3. PC

PC is a measure of linear correlation between two entities [54,55]. Here, correlation between the frequency domain amplitude for the full bandwidth image $y_{FB}$ and band-limited image $y_{model}$ is computed as:

(4) $$\mathrm{PC}=\frac{\mathrm{cov}(y_{FB},y_{model})}{\sigma (y_{FB})}\;\cdot \;\sigma \left(y_{model}\right)$$

where cov represents the covariance and σ represents the standard deviation. PC ∈[−1,1], where 1 denotes complete linear correlation, 0 represents no linear correlation and −1 indicates a perfectly complementary relationship. This metric is used to show the correlation between the frequencies in the obtained reconstruction as compared to the full bandwidth reconstruction.

## 5. Results

### 5.1. Results—20% Bandwidth

**Figure 4:**
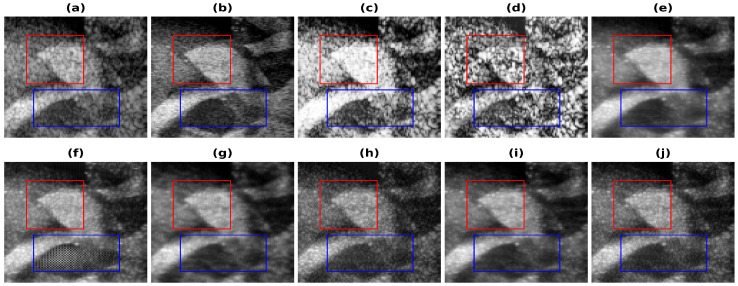
Results of the bandwidth improvement for Phantom I for different models (**a**) Band-limited image (**b**) Full Bandwidth image (**c**) Histogram Equalization (**d**) Contrast Limited Adaptive Histogram Equalization (**e**) U-Net-PL1 (**f**) U-Net-PL2 (**g**) SRCNN-PL1 (**h**) SRCNN-PL2 (**i**) REDNet-PL1 (**j**) REDNet-PL2. Red and blue boxes show the different marked regions.

In Figure 4, the results obtained using Phantom I as test data are presented for different models. Figure 4a shows the band-limited reconstruction obtained after reducing the frequency information to 20%, compared to the full bandwidth reconstruction, which is shown in Figure 4b. The speckle variation is reduced in the full bandwidth reconstruction compared to the band-limited reconstruction. The result obtained using HE and the CLAHE are shown in Figure 4c,d. The reconstruction results obtained using these techniques improve but also over-enhance the contrast and hence lead to information loss. As seen from these reconstructions, the background is also enhanced and hence the RMSE and PSNR values are even higher than the band-limited reconstruction. This is also visible from the increase in the error and decrease in the PSNR values from Table 2. The results obtained using the U-Net model are shown in Figure 4e and Figure 4f, respectively, for the loss functions PL1 and PL2. The reconstruction obtained for Phantom I using SRCNN is shown in Figure 4g and Figure 4h, respectively, for the loss functions PL1 and PL2. The reconstruction obtained using the REDNet is shown in Figure 4i and Figure 4j, respectively, for the loss functions PL1 and PL2. The reconstructions obtained using the PL1 loss are better as compared to using the PL2 loss for training the DL models. The improvements obtained in RMSE and PSNR compared to the band-limited reconstruction are 35.5% and 3.85 dB, utilizing the REDNet model with PL1 loss function.

**Figure 5:**
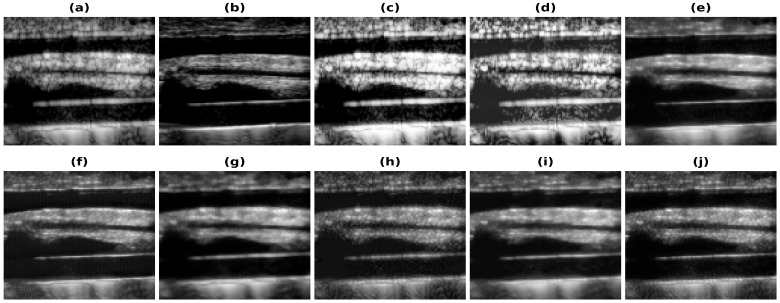
Results of the bandwidth improvement for Carotid artery phantom for different models (**a**) Band-limited image (**b**) Full Bandwidth image (**c**) Histogram Equalization (**d**) Contrast Limited Adaptive Histogram Equalization (**e**) U-Net-PL1 (**f**) U-Net-PL2 (**g**) SRCNN-PL1 (**h**) SRCNN-PL2 (**i**) REDNet-PL1 (**j**) REDNet-PL2.

**Figure 6:**
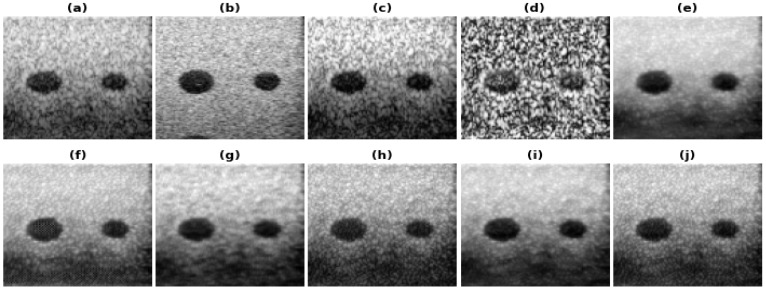
Results of the bandwidth improvement for Commercial phantom for different models (**a**) Band-limited image (**b**) Full Bandwidth image (**c**) Histogram Equalization (**d**) Contrast Limited Adaptive Histogram Equalization (**e**) U-Net-PL1 (**f**) U-Net-PL2 (**g**) SRCNN-PL1 (**h**) SRCNN-PL2 (**i**) REDNet-PL1 (**j**) REDNet-PL2.

**Figure 7:**
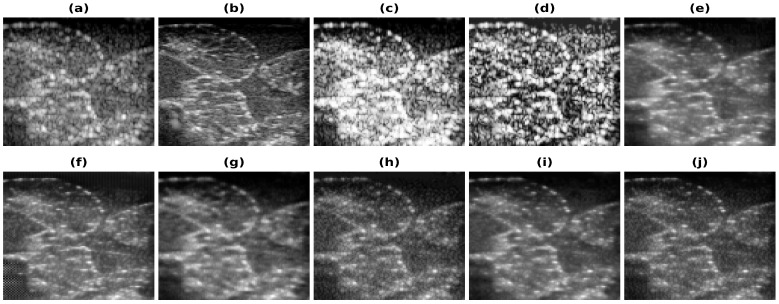
Results of the bandwidth improvement for Phantom II for different models (**a**) Band-limited image (**b**) Full Bandwidth image (**c**) Histogram Equalization (**d**) Contrast Limited Adaptive Histogram Equalization (**e**) U-Net-PL1 (**f**) U-Net-PL2 (**g**) SRCNN-PL1 (**h**) SRCNN-PL2 (**i**) REDNet-PL1 (**j**) REDNet-PL2.

**Figure 8:**
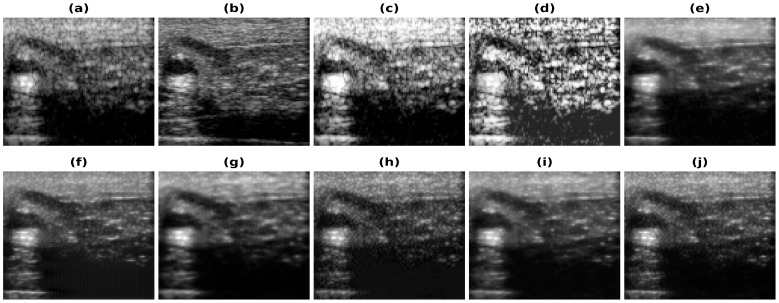
Results of the bandwidth improvement for in vivo carotid mimicking setup for different models (**a**) Band-limited image (**b**) Full Bandwidth image (**c**) Histogram Equalization (**d**) Contrast Limited Adaptive Histogram Equalization (**e**) U-Net-PL1 (**f**) U-Net-PL2 (**g**) SRCNN-PL1 (**h**) SRCNN-PL2 (**i**) REDNet-PL1 (**j**) REDNet-PL2.

Figure 5, Figure 6, Figure 7 and Figure 8 show the reconstructions obtained using the Carotid artery, Commercial phantom, Phantom II, and porcine–pork belly datasets for 20% band-limited data as input. The results and the improvements obtained in RMSE and PSNR compared to the band-limited reconstruction are shown in Table 2 and Table 3, respectively. The REDNet model with PL1 loss performs better in 3 out of 4 cases for these datasets. A Sobel filter is inherently included in the REDNet model, which can increase the features captured by the layers and hence the REDNet model gives the best results among the three models.

The improvements are also visible in the reconstructions obtained, as the artifacts are less and the edges are more visible. The red box shown in Figure 4 shows the improvement in the edge information and less artifacts. The edges are clearer in the full-bandwidth reconstruction shown in Figure 4b as compared to the band-limited reconstruction shown in Figure 4a. The histogram based methods (Figure 4c,d) show a loss in the edge information while the DL based methods show an improvement in the edges and hence improve the reconstruction (Figure 4e–j). The PL1 loss outperforms the PL2 loss in most cases. The reconstruction obtained using the U-Net-PL2 model shows the artifact present in the reconstruction (Figure 4f). The blue box (Figure 4) shows the artifact introduced by the PL2 loss and hence is not suitable for reconstruction. Thus, the PL1 loss is the choice of loss function for the reconstruction although, in some cases the PL2 loss function gives better reconstruction image quality and image metrics.

### 5.2. Results—40% and 60% Bandwidth

Next, we performed the same experiments for the 40% and 60% bandwidth limitation scenarios. The goal of these set of experiments was to investigate whether the model also works for other bandwidth limitations and to check the applicability of the model for different bandwidth reductions. Again, in these cases, the model was trained only on the Phantom I dataset and then tested on all the datasets.

The results obtained when the US images are limited to 40% and 60% bandwidth for all the different test datasets. The RMSE and PSNR values for the 40% and 60% bandwidth reconstruction are provided in Table 2. The improvements obtained for all the test datasets for different bandwidths are shown in Table 3. The reconstructions obtained using the DL-based methods improve the RMSE and PSNR. The DL based reconstructions also reduce the artifacts present in the band-limited reconstructions and thus the image quality is improved. The improvement for 60% bandwidth limitation is lower as compared to the 40 % bandwidth limitation. This occurs since the 60% reconstructions are better than the 40% band-limited reconstructions and this limits the improvement which can be obtained compared to the 40% band-limited reconstructions.

## 6. Discussion/Analysis of the Results

The purpose of this study was to develop and investigate methods based on DL for enhancing the US bandwidth. We evaluated several architectures with different loss functions in their ability to improve the reconstruction. When looking at the metrics, all training pipelines with PL1 loss show an improvement over the bandwidth limited reconstructions, while the PL2 loss does not bring improvement for networks other than REDNet. There is no pipeline that outperforms all others on each metric but in general, the REDNet model with loss PL1 performs best on RMSE and PSNR for all the datasets. The model parameters and model size are shown in Table 4. The REDNet model has the minimum number of parameters as well as the smallest size and gives the best performance for most cases. The developed models and codes are available at https://github.com/navchetan-awasthi/US-Bandwidth-Improvement, accessed on 5 December 2022.

### 6.1. Error Analysis

**Figure 9:**
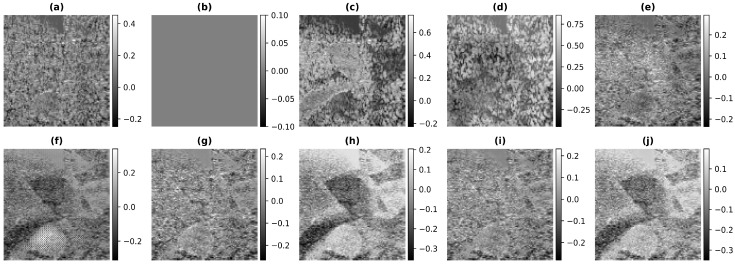
Results of the error of the bandwidth improvement for Phantom I for the different models (**a**) Band-limited image (**b**) Full Bandwidth image (**c**) Histogram Equalization (**d**) Contrast Limited Adaptive Histogram Equalization (**e**) U-Net-PL1 (**f**) U-Net-PL2 (**g**) SRCNN-PL1 (**h**) SRCNN-PL2 (**i**) REDNet-PL1 (**j**) REDNet-PL2.

We have performed an error analysis to see how much the reconstructions obtained using the different methods are different from the full bandwidth reconstruction for Phantom I. In Figure 9, the resulting error between the output image and the target image obtained using Phantom I as the test dataset for different models is presented. Figure 9a is the difference between the full bandwidth reconstruction and the band-limited reconstruction obtained after reducing the frequency information to 20% of that obtained from the full bandwidth reconstruction shown in Figure 4b. The resulting error obtained using the histogram equalization and the contrast limited adaptive histogram equalization are shown in Figure 9c,d. As can be seen from the figures of the resulting error, the dynamic range of values obtained is higher as compared to the band-limited image because of over-enhancement and hence the error is higher in histogram-based methods. The resulting error obtained using the U-Net model are shown in Figure 9e and Figure 9f, respectively, for the different loss functions. The reconstruction error obtained for the Phantom I using SRCNN is shown in Figure 9g and Figure 9h, respectively, for the loss functions PL1 and PL2. The reconstruction obtained using the REDNet is shown in Figure 9i and Figure 9j, respectively, for the loss functions PL1 and PL2. The dynamic range of the error in the colorbar reflects the improvement obtained in the reconstruction as the range is less for DL based methods. Furthermore, loss of structure in the error points to the improvement obtained in the reconstruction. Thus, Figure 9e,g,i show less error as compared to Figure 9f,h,j. This implies that the reconstructions errors obtained using the PL1 loss are low compared to the PL2 loss, which can also be depicted from Table 2.

### 6.2. Frequency Domain Comparison

**Figure 10:**
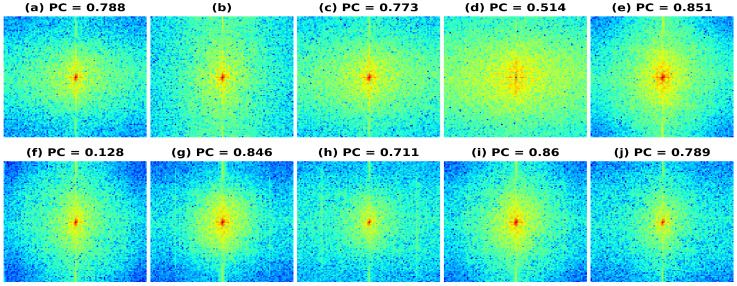
Results of the frequency spectrum of the bandwidth improvement for Phantom II for the different models (**a**) Band-limited image (**b**) Full Bandwidth image (**c**) Histogram Equalization (**d**) Contrast Limited Adaptive Histogram Equalization (**e**) U-Net-PL1 (**f**) U-Net-PL2 (**g**) SRCNN-PL1 (**h**) SRCNN-PL2 (**i**) REDNet-PL1 (**j**) REDNet-PL2.

We also investigated the frequency spectrum response for the reconstruction to understand the improvement in spatial bandwidth of the reconstructions obtained. In Figure 10, the frequency spectrums are plotted using Phantom I reconstructed data as input data for the different models presented. Figure 10a represents the frequency spectrum for the band-limited reconstruction, obtained after reducing the frequency information to 20% of the full bandwidth spectrum (shown in Figure 10b). The resulting frequency spectrum obtained using HE and CLAHE are shown in Figure 10c and Figure 10d, respectively. As seen from the reduction in PC values, there is a reduction in the frequency component correlation and hence the reconstructions obtained have not improved over the band-limited reconstruction. The resulting frequency spectrum obtained using the U-Net, SRCNN, and REDNet are shown in Figure 10e–j. The reconstruction errors obtained using the PL1 loss are low as compared to using the PL2 loss for training the DL models, which is also visible in a high PC value for the PL1 loss and a low PC value for the PL2 loss. The reduction in PC values correlates with the RMSE/PSNR obtained, as can be seen in Table 2. A high RMSE, low PSNR value gives low PC and vice versa and the spectrums of the full bandwidth reconstruction is similar to the reconstructions obtained using DL-based methods with PL1 loss.

### 6.3. Histogram Analysis

**Figure 11:**
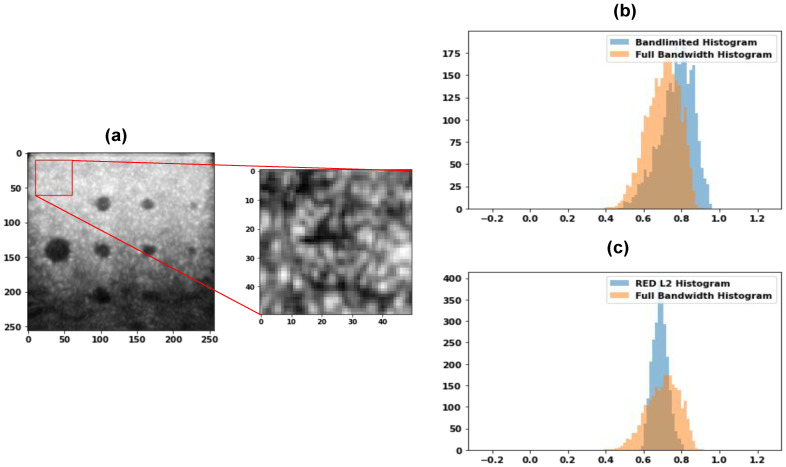
(**a**) Sample of a Commercial phantom image and the corresponding patch area which we want to analyze, (**b**) Comparison of the histogram of band-limited and full bandwidth data, and, (**c**) REDNet PL2 model and full bandwidth histogram.

Finally, we performed first-order speckle statistics, i.e., analyzed the histogram of the speckle for the band-limited data, full bandwidth data, and the images obtained using the DL-based techniques for the envelope image. Figure 11a shows the Commercial phantom used for the speckle characteristics as well as the part of the image used for histogram comparison. The histogram comparison for band-limited and the full bandwidth is shown in Figure 11b, while the histogram comparison for the REDNet PL2 model and the full bandwidth is shown in Figure 11c. As can be seen from the images, the overlap is increased and hence the reconstructed images are better, compared to the band-limited reconstruction; the speckles are more similar in nature, leading to reconstruction near to the full bandwidth reconstruction in visual quality. Next, we performed the analysis of the speckle size. The autocorrelation of the speckle pattern is obtained, and the full width half maximum (FWHM) is computed to obtain the speckle size. The US imaging resolution is anisotropic, and the speckle size is quantitatively evaluated by computing the eccentricity of the ellipse that represents the uniformity of the resolution. The eccentricity of the ellipse containing the longest and the shortest axis is the first eccentricity and is given as:

(5) $$e_{cc_{1}}=\frac{\sqrt{b^{2}-a^{2}}}{b}$$

where *a* and *b* are the shortest and the longest axis.

A uniform distribution of the speckle size results in zero eccentricity of the ellipse, but since the speckle size is disk shaped, a value of zero is impossible to reach, especially at deeper depth. We performed an analysis of the eccentricity of the images before and after the bandwidth improvement. The eccentricity of the ellipse for band-limited image for a set of 10 images was calculated and averaged over the frames and was found to be 0.458 ± 0.073, while for the full bandwidth image it was found to be 0.237 ± 0.205. The eccentricity of the image obtained using the REDNet model was found to be 0.296 ± 0.117 and hence it is improved as compared to the band-limited image. In addition, numerous DL models have been proposed recently for super-resolution enhancement of images, which can also be utilized for improving the bandwidth in US images. An improved residual self-encoding and attention mechanism based super-resolution network was proposed in Ref. [56] and a multi-scale convolution-based model was proposed in Ref. [57]. An efficient model based on neural architecture search and attention mechanism was proposed in Ref. [58]. Other encoder-decoder networks are also available such as binocular rivalry oriented predictive autoencoding network for blind stereoscopic image quality measurement [59] and wavelet-based deep auto encoder-decoder (WDAED)-based image compression [60]. These can also be used for bandwidth improvement after doing some tweaks in the models. All of these models were shown to improve the results for natural images with improved PSNR, and structural similarity index metric (SSIM) and can also be utilized in future research for improving the bandwidth in US images.

### 6.4. Discussion

The models are trained only on the Phantom I dataset and tested on the other datasets. Thus, the model generalizes very well on the unseen datasets and hence can be utilized in real settings without re-training the model. As can be seen from Table 2, the model REDNet-PL1 performs the best in most of the cases with different bandwidth limitations as well as for different test datasets. Thus, REDNet-PL1 model can be utilized for improving the limited bandwidth reconstruction.

The following factors should be considered for potential practical applications of the architectures.

- Firstly, the amount of bandwidth limitation that can be applied to the full bandwidth data. The bandwidth limited reconstructions sometimes may lose too much information for the model to predict certain structures that were present in the original images. This leads to the complete or partial omission of these structures in the model-enhanced reconstructions, and thus a significantly worse metric score, depending on the dataset and the bandwidth limitation.
- Secondly, the models have trouble in predicting the speckle in US images, which leads to smoothed images in some of the results. The loss of speckle also impacts the usability of the images because the speckle is often used for various diagnostic purposes, for example predicting the type of tissue [61]. Most US machines will have some speckle reduction in post processing. So, this should not be a big problem for normal B-mode imaging, if there is improvement in resolution and quality.

For future research, there are some interesting directions that can be considered: Firstly, the models currently use either MSE loss or a combination of VGG16 perceptual loss and MSE. A viable option might be to use other loss functions, for example, using a texture loss as seen in [62] could be considered, because the models currently smooth the images in some cases. A texture loss might result in the generated images being less smoothed and closer to the original texture and thus retain the speckle. Secondly, currently the models cannot predict certain structures due to severe bandwidth limitation. The current models are trained with a 20% fractional bandwidth. A good point of research would be to train a generalized network with all data and different bandwidth limited images as input, to see for what range of bandwidth limitation these structures can or cannot be predicted. Lastly, similar to multi noise level image denoising, where various levels of noise are used as input [63], another interesting approach might be including different bandwidth limitation in the images in the input set to make the models potentially more robust when it comes to different rates of bandwidth limiting.

## 7. Conclusions

In conclusion, DL-based architectures with two different training losses were utilized and compared with histogram-based techniques. The developed methods show that the DL-based methods transform the limited bandwidth reconstruction to an image comparable with one obtained with the full US bandwidth and can thus be utilized for improving reconstruction. We compared some of the state-of-the-art architectures for transforming low bandwidth images into high bandwidth images. The REDNet architecture shows improvements as high as 45.5% on RMSE and 3.85 dB in PSNR for 20% bandwidth limitation. The proposed architecture is lightweight compared to U-Net, has less computational cost, and hence can be utilized in real time settings.

## Figures and Tables

**Table 1:** The number of training, validation and test images for each dataset included in the experiments.

Dataset	Train	Validation	Test
Phantom I	400	135	134
Phantom II	-	-	90
Commercial phantom	-	-	31
Carotid artery	-	-	70
In vivo carotid mimicking setup	-	-	239

**Table 2:** The average metric values for all the test datasets, for all the models and state-of-the-art techniques for 20 % Bandwidth. The best scores for each dataset are written in bold font.

Dataset	Model	20% Bandwidth		40% Bandwidth		60% Bandwidth	
		RMSE	PSNR	RMSE	PSNR	RMSE	PSNR
Phantom I	Bandwidth limited	0.141 ± 0.016	17.049 ± 1.017	0.106 ± 0.012	19.590 ± 1.010	0.080 ± 0.010	21.953 ± 1.111
	HE	0.297 ± 0.044	10.642 ± 1.316	0.280 ± 0.040	11.156 ± 1.296	0.265 ± 0.039	11.632 ± 1.331
	CLAHE	0.344 ± 0.037	9.331 ± 0.948	0.324 ± 0.033	9.838 ± 0.908	0.307 ± 0.032	10.305 ± 0.928
	PL1(U-Net)	0.097 ± 0.012	20.311 ± 1.123	0.134 ± 0.040	17.777 ± 2.387	0.091 ± 0.027	21.132 ± 2.296
	PL2(U-Net)	0.581 ± 0.325	6.140 ± 5.145	0.667 ± 0.246	4.298 ± 4.030	0.083 ± 0.013	21.764 ± 1.328
	PL1(SRCNN)	0.093 ± 0.012	20.683 ± 1.129	0.106 ± 0.011	19.590 ± 1.010	0.062 ± 0.009	24.276 ± 1.296
	PL2(SRCNN)	0.131 ± 0.015	17.690 ± 0.915	0.108 ± 0.016	19.384 ± 1.208	0.080 ± 0.013	22.014 ± 1.428
	PL1(RED)	**0.091 ± 0.012**	**20.903 ± 1.189**	**0.077 ± 0.010**	**22.369 ± 1.219**	**0.059 ± 0.008**	**24.661 ± 1.302**
	PL2(RED)	0.116 ± 0.013	18.802 ± 0.956	0.092 ± 0.014	20.839 ± 1.279	0.072 ± 0.012	22.978 ± 1.442
Carotid artery	Bandwidth limited	0.165 ± 0.026	15.768 ± 1.376	0.110 ± 0.018	19.266 ± 1.359	0.084 ± 0.013	21.577 ± 1.327
	HE	0.313 ± 0.030	10.131 ± 0.856	0.269 ± 0.026	11.451 ± 0.862	0.242 ± 0.023	12.348 ± 0.836
	CLAHE	0.344 ± 0.028	9.310 ± 0.733	0.298 ± 0.026	10.557 ± 0.761	0.270 ± 0.023	11.395 ± 0.753
	PL1(U-Net)	0.096 ± 0.015	20.446 ± 1.378	0.236 ± 0.020	12.560 ± 0.757	0.169 ± 0.018	15.490 ± 0.901
	PL2(U-Net)	0.247 ± 0.121	13.010 ± 3.674	0.806 ± 0.088	1.927 ± 0.955	0.088 ± 0.005	21.120 ± 0.512
	PL1(SRCNN)	0.107 ± 0.013	19.477 ± 1.162	0.080 ± 0.012	22.002 ± 1.318	0.066 ± 0.010	23.657 ± 1.381
	PL2(SRCNN)	0.151 ± 0.007	16.435 ± 0.408	0.112 ± 0.008	19.028 ± 0.606	0.082 ± 0.008	21.786 ± 0.890
	PL1(RED)	**0.095 ± 0.013**	**20.523 ± 1.242**	**0.077 ± 0.011**	**22.399 ± 1.333**	**0.063 ± 0.010**	**24.134 ± 1.471**
	PL2(RED)	0.125 ± 0.008	18.110 ± 0.608	0.093 ± 0.009	20.655 ± 0.858	0.074 ± 0.008	22.716 ± 0.960
Commercial phantom	Bandwidth limited	0.204 ± 0.032	13.885 ± 1.276	0.195 ± 0.040	14.390 ± 1.724	0.187 ± 0.049	14.874 ± 2.363
	HE	0.236 ± 0.024	12.586 ± 0.840	0.229 ± 0.030	12.863 ± 1.130	0.226 ± 0.035	13.039 ± 1.366
	CLAHE	0.287 ± 0.023	10.871 ± 0.674	0.282 ± 0.027	11.017 ± 0.823	0.279 ± 0.031	11.130 ± 0.967
	PL1(U-Net)	**0.197 ± 0.020**	14.146 ± 0.884	0.210 ± 0.032	13.638 ± 1.359	0.187 ± 0.041	14.814 ± 2.115
	PL2(U-Net)	0.421 ± 0.095	7.736 ± 1.983	0.467 ± 0.070	6.720 ± 1.340	0.338 ± 0.357	12.348 ± 6.212
	PL1(SRCNN)	0.202 ± 0.025	13.956 ± 1.110	0.192 ± 0.018	14.354 ± 0.800	0.183 ± 0.032	14.876 ± 1.600
	PL2(SRCNN)	0.223 ± 0.063	13.429 ± 2.717	0.217 ± 0.045	13.483 ± 1.915	0.188 ± 0.030	14.623 ± 1.338
	PL1(RED)	0.201 ± 0.025	13.985 ± 1.120	**0.191 ± 0.019**	14.410 ± 0.844	0.182 ± 0.037	14.968 ± 1.866
	PL2(RED)	0.204 ± 0.061	**14.242 ± 2.894**	0.193 ± 0.047	**14.575 ± 2.238**	**0.179 ± 0.026**	**15.013 ± 1.217**
Phantom II	Bandwidth limited	0.155 ± 0.021	16.297 ± 1.212	0.100 ± 0.012	20.044 ± 1.132	0.074 ± 0.009	22.690 ± 1.201
	HE	0.318 ± 0.032	9.994 ± 0.936	0.282 ± 0.025	11.021 ± 0.815	0.260 ± 0.022	11.742 ± 0.778
	CLAHE	0.355 ± 0.027	9.017 ± 0.672	0.317 ± 0.023	10.007 ± 0.634	0.294 ± 0.022	10.672 ± 0.662
	PL1(U-Net)	0.089 ± 0.012	21.086 ± 1.217	0.207 ± 0.040	13.869 ± 1.874	0.146 ± 0.030	16.932 ± 1.910
	PL2(U-Net)	0.393 ± 0.210	9.334 ± 4.655	0.854 ± 0.173	1.566 ± 1.929	0.081 ± 0.006	21.862 ± 0.669
	PL1(SRCNN)	0.096 ± 0.011	20.427 ± 1.067	0.069 ± 0.010	23.331 ± 1.248	0.054 ± 0.008	25.395 ± 1.270
	PL2(SRCNN)	0.144 ± 0.013	16.855 ± 0.767	0.106 ± 0.009	19.513 ± 0.709	0.074 ± 0.009	22.646 ± 0.984
	PL1(RED)	**0.085 ± 0.012**	**21.457 ± 1.238**	**0.065 ± 0.010**	**23.790 ± 1.368**	**0.051 ± 0.007**	**25.975 ± 1.274**
	PL2(RED)	0.119 ± 0.009	18.506 ± 0.630	0.087 ± 0.009	21.245 ± 0.899	0.067 ± 0.009	23.577 ± 1.099
In vivo carotid mimicking setup	Bandwidth limited	0.172 ± 0.040	15.487 ± 1.876	0.134 ± 0.027	17.591 ± 1.695	0.110 ± 0.018	19.274 ± 1.482
	HE	0.276 ± 0.042	11.271 ± 1.303	0.254 ± 0.039	11.984 ± 1.302	0.244 ± 0.036	12.361 ± 1.285
	CLAHE	0.302 ± 0.038	10.482 ± 1.090	0.276 ± 0.036	11.257 ± 1.113	0.266 ± 0.033	11.554 ± 1.065
	PL1(U-Net)	0.143 ± 0.027	17.081 ± 1.680	0.250 ± 0.028	12.103 ± 0.976	0.177 ± 0.023	15.127 ± 1.134
	PL2(U-Net)	0.199 ± 0.078	14.522 ± 2.834	0.736 ± 0.087	2.723 ± 0.995	0.100 ± 0.013	20.094 ± 1.097
	PL1(SRCNN)	0.135 ± 0.023	17.558 ± 1.544	0.117 ± 0.019	18.775 ± 1.477	0.100 ± 0.016	20.134 ± 1.487
	PL2(SRCNN)	0.148 ± 0.013	16.602 ± 0.719	0.130 ± 0.017	17.784 ± 1.130	0.104 ± 0.016	19.795 ± 1.400
	PL1(RED)	0.133 ± 0.022	17.654 ± 1.536	0.118 ± 0.019	18.710 ± 1.476	0.098 ± 0.016	20.340 ± 1.553
	PL2(RED)	**0.130 ± 0.015**	**17.787 ± 0.975**	**0.111 ± 0.017**	**19.204 ± 1.316 **	**0.096 ± 0.015**	**20.485 ± 1.424**

**Table 3:** The improvements obtained for all test datasets with the different models and state-of-the-art techniques for 20%, 40% and 60% Bandwidth.

Dataset	Bandwidth	RMSE (%)	PSNR (dB)
Phantom I	20%	35.46	3.85
	40%	27.35	2.77
	60%	26.25	2.70
Carotid artery	20%	42.42	4.76
	40%	30.00	3.13
	60%	25.00	2.56
Commercial phantom	20%	3.43	0.36
	40%	2.05	0.18
	60%	4.28	0.14
Phantom II	20%	45.16	5.16
	40%	35.00	3.75
	60%	31.08	3.28
In vivo carotid mimicking setup	20%	24.24	2.30
	40%	17.16	1.61
	60%	12.72	1.21

**Table 4:** Total parameters including the Model parameters (Trainable Parameters and Non-trainable Parameters) and Model Size are shown for all the models.

Model	Total Parameters	Trainable Parameters	Non-Trainable Parameters	Model Size (MB)
SRCNN	85,889	85,889	0	1.06
U-Net	2,164,433	2,161,489	2944	26.3
REDNet	60,760	60,440	320	0.87

## Data Availability

Not applicable.
